# Adolescent Social Defeat Induced Alterations in Social Behavior and Cognitive Flexibility in Adult Mice: Effects of Developmental Stage and Social Condition

**DOI:** 10.3389/fnbeh.2016.00149

**Published:** 2016-07-20

**Authors:** Fan Zhang, Sanna Yuan, Feng Shao, Weiwen Wang

**Affiliations:** ^1^Key Laboratory of Mental Health, Institute of Psychology, Chinese Academy of SciencesBeijing, China; ^2^The University of Chinese Academy of SciencesBeijing, China; ^3^Department of Psychology and Beijing Key Laboratory of Behavior and Mental Health, Peking UniversityBeijing, China

**Keywords:** social defeat stress, adolescent, social condition, social avoidance, cognitive flexibility

## Abstract

Negative social experiences during adolescence increase the risk of psychiatric disorders in adulthood. Using “resident-intruder” stress, the present study aimed to investigate the effects of adolescent social defeat on emotional and cognitive symptoms associated with psychiatric disorders during adulthood and the effects of the developmental stage and social condition on this process. In Experiment 1, animals were exposed to social defeat or manipulation for 10 days during early adolescence (EA, postnatal days [PND] 28–37), late adolescence (LA, PND 38–47), and adulthood (ADULT, PND 70–79) and then singly housed until the behavioral tests. Behaviors, including social avoidance of the defeat context and cortically mediated cognitive flexibility in an attentional set-shifting task (AST), were assessed during the week following stress or after 6 weeks during adulthood. We determined that social defeat induced significant and continuous social avoidance across age groups at both time points. The mice that experienced social defeat during adulthood exhibited short-term impairments in reversal learning (RL) on the AST that dissipated after 6 weeks. In contrast, social defeat during EA but not LA induced a delayed deficit in extra-dimensional set-shifting (EDS) in adulthood but not during adolescence. In Experiment 2, we further examined the effects of social condition (isolation or social housing after stress) on the alterations induced by social defeat during EA in adult mice. The adult mice that had experienced stress during EA exhibited social avoidance similar to the avoidance identified in Experiment 1 regardless of the isolation or social housing after the stress. However, social housing after the stress ameliorated the cognitive flexibility deficits induced by early adolescent social defeat in the adult mice, and the social condition had no effect on cognitive function. These findings suggest that the effects of social defeat on emotion and cognitive function are differentially affected by the developmental stage and social condition. EA may comprise a particularly sensitive developmental period in which social defeat may produce a delayed impairment in cognitive flexibility during adulthood, and the social condition following stress appears to play an important intermediary role in the development of these cognitive deficits.

## Introduction

In humans, bullying and subordination are prevalent stressors throughout life and are strongly related to the onset of psychiatric disorders (Brown and Prudo, 1981; Taylor et al., 2011). These disorders are clinically heterogeneous with distinct symptoms that reflect emotional and cognitive dysfunctions. The impairment of cognitive flexibility, which comprises the ability to adapt to dynamic environments using appropriate behavioral strategies in which the prefrontal cortex (PFC) plays an integrative role, is extensively present in psychiatric disorders, such as depression (Lapiz-Bluhm et al., 2008). Human bulling experiences may be effectively modeled by social defeat in rodents using the resident-intruder paradigm (Miczek, 1979; Golden et al., 2011). Numerous studies have demonstrated that repeated exposure to social defeat elicits a set of depressive and anxious behaviors in adult rodents, including anhedonia, anxiety in the elevated plus maze and open field, and social avoidance (Buwalda et al., 2005; Venzala et al., 2012). Previous studies from our group and other groups have demonstrated that chronic social defeat also induced deficits in cognitive flexibility in adult Sprague-Dawley rats (Wang et al., 2012; Snyder et al., 2015). In many cases, the behavioral alterations induced by adult social defeat are not lasting and are reversible following a period of recovery (however, fear memories of the defeat context may last longer; Buwalda et al., 2005; Venzala et al., 2012; Snyder et al., 2015).

The developmental stage in which the exposure to a stressor occurs strongly influences the behavioral effects of the stress (Lupien et al., 2009). In adolescence, a transition period between childhood and adulthood that occurs in both humans and rodents, the ongoing development of the structure and function of neural systems implicated in emotion and cognition, particularly the PFC and relevant pathways, may render adolescents more or differentially susceptible to stress compared with adults (Spear, 2000; McCormick and Green, 2013). For example, the structures of the PFC, including the synapses and receptor expression, undergo profound development throughout adolescence, with peak increases during early adolescence (EA, postnatal days [PND] 28–31) and subsequent decreases to adult levels (Andersen and Teicher, 2008; Tamnes et al., 2010). The neural connections between the medial prefrontal cortex (mPFC) and the subcortical limbic regions, such as the amygdala and hippocampus, also extensively change during adolescence. In addition, the hypothalamic-pituitary-adrenal (HPA) axis remains immature during adolescence (Andersen, 2003). For example, adolescents often exhibit an enhanced or prolonged HPA response to stressors compared with adults (McCormick et al., 2010). Therefore, the developmental trajectory of these neural systems may be particularly vulnerable to stress during adolescence, with long-term behavioral consequences (Andersen and Teicher, 2008; Leussis et al., 2008; Morrissey et al., 2011; McCormick and Green, 2013).

Social conditions (isolation or social housing) may confound and interact with stress effects. In adult rodents, isolation and social housing following defeat aggravate or ameliorate, respectively, the behavioral and physiological changes induced by social defeat (de Jong et al., 2005; Buwalda et al., 2011; McCormick and Green, 2013). For example, a single social defeat followed by isolation housing produces more lasting alterations in anxious behaviors in the elevated plus maze and open field tests compared with social housing following defeat (Ruis et al., 1999; Nakayasu and Ishii, 2008). Social housing relieves the effects of social defeat on the heart rate, body temperature and loss of body weight compared with isolation housing (Meerlo et al., 1999; de Jong et al., 2005). In addition, adolescence is a sensitive period in terms of the social conditions (Lukkes et al., 2009; Eiland and Romeo, 2013). We and other groups have demonstrated that isolation housing during adolescence induces greater and/or more lasting changes in various behaviors, including latent inhibition, spatial cognition, and anxiety (Leussis and Andersen, 2008; Lukkes et al., 2009; Shao et al., 2009).

Overall, the present study aimed to investigate the developmental profile of the emotional and cognitive dysregulations induced by adolescent social defeat and the effects of the developmental stage and social condition on this process. In Experiment 1, the short- and long-term effects of repeated social defeat (for 10 days, followed by isolation housing) during EA (PND 28–37), late adolescence (LA, PND 38–47), and adulthood (ADULT, PND 70–79) on the social avoidance and cognitive flexibility of mice were determined during the week following the last stress experience and after 6 weeks in adulthood. Social avoidance was assessed as an emotional measure of a specific anxiety regarding the defeat context (Golden et al., 2011). Cognitive flexibility was assessed in the attentional set-shifting task (AST), a well-characterized task used to systematically assess different cognitive components in rodents (Birrell and Brown, 2000; Liston et al., 2006). Lesion and functional studies have utilized the AST to demonstrate that the core components of cognitive flexibility, reversal learning (RL) and extra-dimensional set-shifting (EDS) depend on the functional integrity of the orbitofrontal cortex (OFC) and the mPFC (Birrell and Brown, 2000). Chronic stressors may impair different components of cognitive flexibility as a result of the structural and functional changes in the PFC (Liston et al., 2006; Lapiz-Bluhm et al., 2009; Bondi et al., 2010). Therefore, comparisons of the performances in the different cognitive components of the AST may comprise an indirect measure of the integrity of the underlying brain regions. In rodents, a conservative definition of adolescence spans PND 28–48 (Spear, 2000; McCormick et al., 2010). In preliminary research, we demonstrated that social defeat for 10 days (PND 70–79) induces deficits in RL in the AST in adult mice tested during the following week after the stress (data shown in Experiment 1). Thus, adolescent stress was delivered during EA (PND 28–37) and LA (PND 38–47). Rodents in these two stages exhibited different behavioral and physiological responses to stress (McCormick et al., 2010; Bingham et al., 2011; Schneider, 2013). Based on the results of Experiment 1, Experiment 2 further investigated the effects of the social condition after defeat on adult changes induced by early adolescent social defeat.

## Materials and Methods

### Animals

The intruder experimental subjects comprised male offspring of C57BL/6J mice (the Academy of Chinese Military Medical Science) obtained at weaning (PND 21) from our in-house breeding program (Center of Experimental Animal, Institute of Psychology, Chinese Academy of Sciences). The resident compound discrimination (CD)-1 mice (2 months old, Vital River Laboratories) were individually housed. All animals were maintained at 22°C on a 12-h light/dark cycle (lights on at 07:00 h) with free access to food and water with the exception of during the AST test. All procedures were approved by the Institutional Review Board of the Institute of Psychology, Chinese Academy of Sciences.

### Experimental Procedures

Two experiments were conducted in the present study.

**Figure 1:**
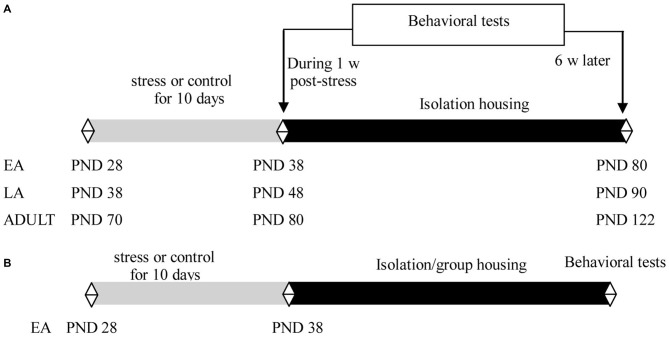
**Experimental design and timeline of procedures in Experiments 1 (A) and 2 (B)**.

Experiment 1 was performed using 21 day-old male offspring of C57BL/6J mice. The mice were socially housed with 2–4 littermates per cage until the start of the experiment. Littermates in EA (PND 28), LA (PND 38), or adulthood (PND 70) were assigned to stress and control groups. The mice were exposed to 10 consecutive days of social defeat or control manipulation. Half of the mice in each group (9–11 mice/group in each age) were subsequently subjected to behavioral tests during the week after the last exposure to stress; the remaining mice (9–11 mice/group in each age) were individually housed for 6 weeks for behavioral tests (Figure 1A).

Experiment 2 included 21 day-old male offspring of C57BL/6J mice. The mice were socially housed with 2–4 littermates per cage until the start of the experiment. Littermates at PND 28 were assigned to stress and control groups. The mice were exposed to 10 consecutive days of social defeat or control manipulation. After the last stress, the mice in each group were further assigned to isolation or social housing subgroups, in which the mice were individually housed (9–12 mice/group) or returned to their familiar groups (9–12 mice/group), respectively. To minimize the effects of changing cage mates and aggression among the group-housed male mice, which may have detrimental consequences for emotion and cognitive function (McQuaid et al., 2013; McCormick et al., 2015), the group-housed male mice comprised siblings and were maintained in the same group before and after the social defeat in the present study. Behavioral tests were performed after 6 weeks during adulthood (Figure 1B).

### Stress Regimen

For social defeat, an adaptation of the previously described resident-intruder protocol was used (Golden et al., 2011). CD-1 mice (potential aggressors) were placed in the experimental cages for at least 7 days to encourage territoriality. Three days prior to the social defeat procedure, the CD-1 mice were selected as aggressors (residents) based on their attack latencies (shorter than 60 s on two consecutive sessions) during three screening tests (180 s each, once daily, direct exposure to a new C57BL/6J mouse each time). The experimental cages of the resident CD-1 mice were divided into two compartments using a clear perforated Plexiglas divider, and the resident was placed into one compartment 24 h prior to the start of the defeat session. On the first day of social defeat, a single C57BL/6J mouse from the social defeat group (intruder) was placed in the vacant compartment of the cage and directly exposed to the resident mouse by removing the divider. Direct physical contact between the resident and intruder continued for 5 min or 3 min if the attack by the resident was intense. The animals were subsequently separated by the divider; however, they maintained olfactory and visual contact for the remainder of the day. The intruders were rotated every day for nine subsequent defeat days to avoid habituation to a single aggressor. C57BL/6J control mice were housed in pairs with members of the same strain and were maintained on opposite sides of the divider. All control mice were rotated on a daily basis in a manner similar to the mice that underwent defeat; however, they were never allowed physical contact with their cage mate.

### Behavioral Tests

#### Social Avoidance

Social avoidance towards a novel CD-1 mouse was assessed in a two-stage social interaction test as previously described (Golden et al., 2011). The arena comprised an opaque plastic open field (40 × 40 × 40 cm) that contained an empty metallic mesh cage (8 × 8 × 8 cm) secured at one end of the field. C57BL/6J experimental mice were habituated to the testing suite for 1 h prior to testing. Testing occurred under red-light conditions in a room isolated from external sound sources. Each experimental mouse was introduced into the open field, and its trajectory was tracked in two consecutive sessions (2.5 min per session with an interval of 30 s) with or without the target CD-1 mouse present in the metallic mesh cage. The time spent by the experimental mouse in the “interaction zone” (the corridor 8 cm wide that surrounded the metallic cage) during each session was recorded and analyzed using Noldus EthoVision Software (EthovisionXT with Social Interaction Module; Noldus Information Technology). Social avoidance was measured using the social interaction ratio, which was calculated as the time in the interaction zone with target CD-1/time in the interaction zone without target CD-1. After each test, the arena was cleaned with 75% ethanol to prevent olfactory interference with subsequent tests.

#### AST

The AST was initiated after the social avoidance test. The testing box comprised a white rectangular Plexiglas arena (L × W × H: 30 × 26 × 20 cm) that was divided into a starting arena and a testing arena using a removable panel. For each trial, the animal was maintained in the starting area until the divider was removed. The mice were trained to locate a food reward (1/8 of a Honey Nut O manufactured by Nhong Shim Kellogg, Co., Ltd.) in two ceramic pots (inner diameter 3.5 cm; depth 3 cm), which were marked by cues in two dimensions: the digging medium within the pot and the odor applied to the pot.

The methods were adapted from previous studies by our laboratory and other groups (Bissonette and Powell, 2012; Yuan et al., 2014). Briefly, the mice were moderately food-restricted (2.5–3.5 g/day) for 1 week to maintain 80–85% of the original body weight with water freely available. A 4-day behavioral protocol was initiated on the third day of food restriction. On day 1, the mice were trained to retrieve a fully buried food reward by digging in two sawdust-filled pots placed in the home cage until each retrieval was completed within 5 min in three consecutive trials. On day 2, the mice were transferred to the testing arena and trained to retrieve the reward from both sawdust-filled pots within 5 min for three consecutive trials. On day 3, the mice were trained to perform two separate simple discriminations (SDs) using two sets of exemplar pots scented with different odors (lemon vs. rosewood, both pots filled with sawdust) or filled with different digging media (foam sheets vs. shredded paper, no odor) until the criterion of six consecutive correct trials was met. On day 4, the mice were tested on a series of five increasingly difficult discriminations until the criterion of six consecutive correct trials was met. An example of the task sequence is presented in Table 1. In the first stage of SD, the mice were required to discriminate between two media (scouring pad and facial puff), only one of which (e.g., scouring pad) predicted the food reward. In the CD stage, the same discrimination was required as the SD; however, irrelevant stimuli (rosemary and nutmeg) in a new dimension (odor) were introduced in this stage. In the intra-dimensional shifting (IDS) stage, two new exemplars from each dimension were introduced; however, the medium remained the relevant dimension. In the RL stage, the same odors and media were used, and the medium remained the relevant dimension; however, the negative medium in the IDS was positive (e.g., googly eyes) and the previously positive medium was negative (e.g., wooden ball). In the stage of EDS, two new exemplars from each dimension were introduced, and the relevant dimension was changed from medium to odor. All mice were tested using the same pairs of exemplars in the same order; the assignment of each exemplar in a pair as positive or negative in a given stage and the left-right positioning of the pots in the arena in each trial were determined randomly in advance. The number of trials required to reach the criterion of six consecutive correct responses and the number of errors at each stage were recorded.

**Table 1:** **Testing protocol for five-stage attentional set-shifting task (AST)**.

Discrimination stage	Dimensions		Example combinations	
	Relevant	Irrelevant	(+)	(−)
SD	Medium		**Scouring pad**	Facial puff
CD	Medium	Odor	**Scouring pad**/Rosemary	Facial puff/Nutmeg
			**Scouring pad**/Nutmeg	Facial puff/Rosemary
IDS	Medium	Odor	**Wooden balls**/Clove	Googly eyes/Cinnamon
			**Wooden balls**/Cinnamon	Googly eyes/Clove
RL	Medium	Odor	**Googly eyes**/Clove	Wooden balls/Cinnamon
			**Googly eyes**/Cinnamon	Wooden balls/Clove
EDS	Odor	Medium	**Citronella**/White paper	Thyme/Crepe paper
			**Citronella**/Crepe paper	Thyme/White paper

*Examples of stimulus pairs and the progression through stages of the AST protocol. For each stage, the positive stimulus in bold was randomly paired across trials with the two stimuli from the irrelevant dimension. The assignment of each exemplar in a pair as positive or negative in a given stage, as well as the left-right positioning of the pots in the arena on each trial, were randomly determined in advance*.

### Statistical Analysis

The commercially available program SPSS 16.0 was used for the statistical analysis. Data are presented as the mean ± standard error of the mean (SEM) for all measures. Social avoidance data were analyzed by 2-way analysis of variance (ANOVA; stress × age in Experiment 1 and stress × social condition in Experiment 2). The AST data were analyzed by 3-way ANOVA (stress × age × stage in Experiment 1 and stress × social condition × stage in Experiment 2) with repeated measures over stages. Where significant main effects or interactions were indicated, a *post hoc* analysis was conducted. Significance for all analyses was determined as *p* < 0.05. To assess the validity of the AST task, the data for each AST test in the control mice were used to analyze the effect of the stage. As previously reported by Birrell and Brown (2000) and Lapiz-Bluhm et al. (2008), the relatively increased difficulty in the RL and EDS stages demonstrate the validity of the strategic and extra-dimensional attentional shifting manipulation. Consistently, the present study demonstrated that a significant main effect of stage existed on each AST performance in the control mice, which was indicated by increased numbers of trials and/or errors to the criterion in the RL and/or EDS stages compared with the other stages; these findings suggest that the AST used in the present study is validated. To avoid repetition, the validity analysis was not discussed in the subsequent section.

## Results

### Experiment 1—Effects of Social Defeat on Social Avoidance in Mice of Different Ages

#### Short-Term Effects

**Figure 2:**
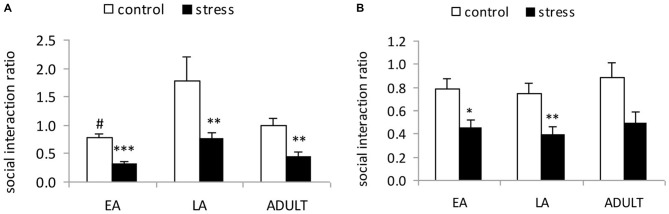
**Short-term and long-term effects of social defeat on social avoidance in mice of different ages (Mean/SEM).** The bars indicate the mean social interaction ratios, calculated as the ratio of the time spent in the interaction zone with the target compound discrimination (CD)-1 mice to the time spent in the interaction zone without the target CD-1 mice. **(A)** Mice were tested shortly (1 day) after the last stress exposure. **(B)** Mice were tested after 6 weeks. ^#^*p* < 0.05 compared with the late adolescent controls. *p < 0.05, **p < 0.01, ***p < 0.001 compared with the corresponding age controls.

A 2-way ANOVA (stress × age) indicated significant main effects of age (*F*_(2,53)_ = 7.107, *p* = 0.002) and stress (*F*_(1,53)_ = 17.129, *p* < 0.001); however, there was no significant stress × age interaction (*F*_(2,53)_ = 2.111, *p* = 0.357; Figure 2A). The *post hoc* analysis in the control mice indicated that the social interaction ratio of the LA mice was significantly higher than the EA mice (*p* = 0.02) and moderately higher than the ADULT mice (*p* = 0.072). Social defeat induced social avoidance in all three age groups, which was indicated by a significantly lower social interaction ratio relative to the respective control mice (EA: *p* < 0.0001; LA: *p* = 0.0037; and ADULT: *p* = 0.0013).

#### Long-Term Effects

A 2-way ANOVA (stress × age) indicated a significant main effect of stress (*F*_(1,56)_ = 8.563, *p* = 0.005); however, there was no main effect of age (*F*_(2,56)_ = 0.291, *p* = 0.749) or an interaction between age and stress (*F*_(2,56)_ = 1.668, *p* = 0.199; Figure 2B). Social defeat induced social avoidance in all three age groups, which was indicated by a significantly lower social interaction ratio in the adult mice stressed during EA (*p* < 0.05) and LA (*p* < 0.01), as well as a tendency towards a reduction in the social interaction ratio in the mice stressed during adulthood compared with the corresponding age controls (*p* = 0.052).

### Experiment 1—Effects of Social Defeat on the AST Performance of Mice of Different Ages

#### Short-Term Effects

**Figure 3:**
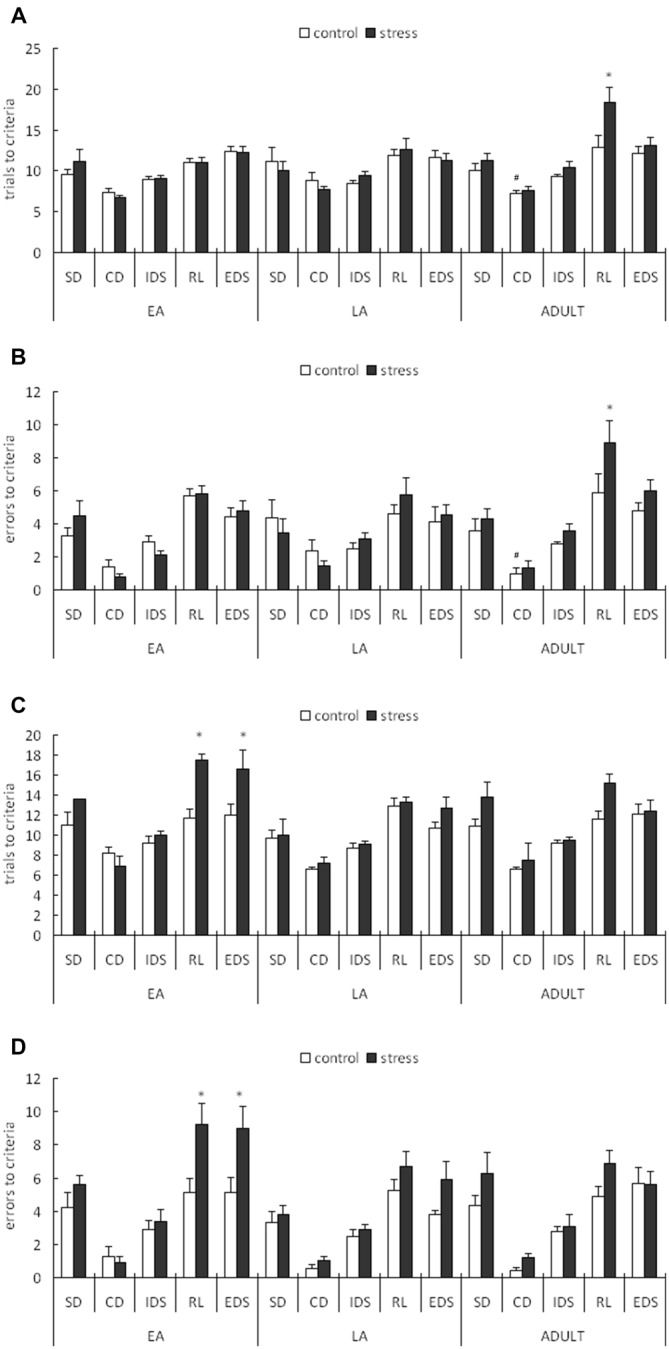
**Short-term and long-term effects of social defeat on the performance of the attentional set-shifting task (AST) in mice of different ages (Mean/SEM).** The AST was administered during the week following stress **(A,B)** and after 6 weeks **(C,D)**. The bars represent the means of 9–11 rats per group. *p < 0.05 compared with the adult controls; ^#^*p* < 0.05 compared with the late adolescent controls. SD, simple discrimination; CD, compound discrimination; IDS, intra-dimensional shift; RL, reversal learning; EDS, extra-dimensional set-shifting.

For the trials to the criterion tested during the week following stress exposure, a 3-way ANOVA (stress × age × stage) indicated significant main effects of stage (*F*_(4,200)_ = 46.258, *p* < 0.0001) and age (*F*_(2,50)_ = 3.696, *p* = 0.032) but not stress (*F*_(1,50)_ = 2.4, *p* = 0.128). There was also a significant stage × age interaction (*F*_(8,200)_ = 2.229, *p* = 0.027; Figure 3A). A *post hoc* analysis of the stage × age interaction in the control mice indicated that more trials were required to learn the CD task in the late adolescent mice compared with the adult mice (*p* = 0.02) and early adolescent mice (*p* = 0.071). An ANOVA for all tasks identified a significant stress × age interaction only for the RL stage (*F*_(2,50)_ = 3.231, *p* = 0.048). No other main or interaction effects were identified for the other stages. *Post hoc* comparisons for the RL stage indicated that the mice stressed during adulthood required more trials to learn the RL task compared with the corresponding age controls (*p* = 0.005), whereas there was no difference between the control and stressed groups of the early and late adolescent mice (*p* = 0.903 for EA; *p* = 0.610 for LA).

For the errors before the criterion tested during the week following stress exposure, a 3-way ANOVA (stress × age × stage) indicated a significant main effect of stage (*F*_(4,200)_ = 57.002, *p* < 0.0001) but not age (*F*_(2,50)_ = 1.832, *p* = 0.171) or stress (*F*_(1,50)_ = 2.047, *p* = 0.159). There was also a marginal stage × age interaction (*F*_(8,200)_ = 1.813, *p* = 0.077; Figure 3B). A *post hoc* analysis of the stage × age interaction in the control mice indicated that slightly more failures before the criterion in the CD stage were required in the late adolescent mice compared with the adult mice (*p* < 0.05). An ANOVA for all task stages identified a marginally significant stress effect only for the RL stage (*F*_(1,50)_ = 3.707, *p* = 0.060). No other main or interaction effects were identified in the other test stages. *Post hoc* comparisons for the RL stage indicated that the mice stressed during adulthood exhibited more failures before the criterion compared with the corresponding control mice (*p* = 0.0336), whereas no difference was identified between the control and stressed groups of the early and late adolescent mice (*p* = 0.938 for EA; *p* = 0.455 for LA).

#### Long-Term Effects

For the trials to criterion tested after 6 weeks during adulthood, a 3-way ANOVA (stress × age × stage) indicated significant main effects of stage (*F*_(4,224)_ = 51.307, *p* < 0.0001), age (*F*_(2,56)_ = 4.986, *p* = 0.01) and stress (*F*_(1,56)_ = 14.417, *p* < 0.0001). There was also a significant stage × stress interaction (*F*_(4,224)_ = 2.922, *p* = 0.022; Figure 3C). A subsequent ANOVA for each stage indicated significant stress effects for RL (*F*_(1,56)_ = 9.039, *p* = 0.004) and EDS (*F*_(1,56)_ = 6.723, *p* = 0.012). *Post hoc* comparisons demonstrated that the adult mice stressed during EA exhibited higher trials to the criterion for RL (*p* = 0.018) and EDS (*p* = 0.035) compared with the control mice of the same age.

For the errors before the criterion tested after 6 weeks during adulthood, a 3-way ANOVA (stress × age × stage) indicated significant main effects of stage (*F*_(4,224)_ = 59.448, *p* < 0.0001), age (*F*_(2,56)_ = 4.187, *p* = 0.049) and stress (*F*_(1,56)_ = 11.779, *p* = 0.001). There was also a significant stage × stress interaction (*F*_(4,224)_ = 2.612, *p* = 0.036; Figure 3D). A subsequent ANOVA for each stage indicated significant stress effects for RL (*F*_(1,56)_ = 11.177, *p* = 0.001) and EDS (*F*_(1,56)_ = 7.754, *p* = 0.007). *Post hoc* comparisons demonstrated that the adults stressed during EA exhibited more failures before the criterion on the RL (*p* = 0.014) and EDS (*p* = 0.027) tasks compared with the control mice of the same age.

### Experiment 2—Effects of Social Condition on the Alteration of Social Avoidance in Adult Mice Induced by Early Adolescent Social Defeat

**Figure 4:**
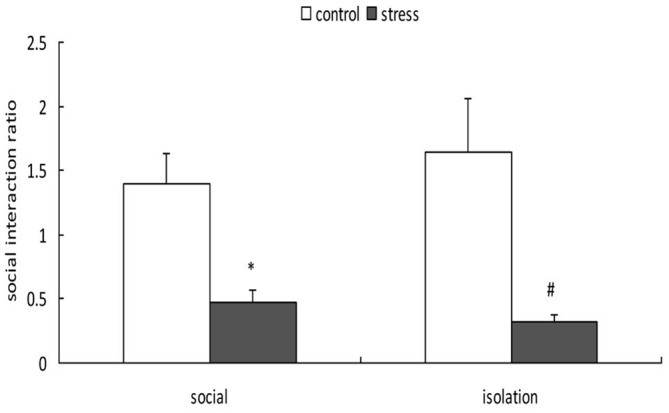
**Effects of social condition on early adolescent social defeat-induced alterations in social avoidance in adult mice (Mean/SEM).** *p < 0.05 compared with the control group with social housing; ^#^*p* < 0.05 compared with the control group with isolation housing.

A 2-way ANOVA (stress × social condition) for social avoidance indicated a significant main effect of stress (*F*_(1,37)_ = 24.803, *p* < 0.01); however, there was no main effect of social condition (*F*_(1,37)_ = 0.048, *p* = 0.828) or an interaction between stress and social condition (*F*_(1,37)_ = 0.761, *p* = 0.389; Figure 4). Exposure to social defeat during EA produced significantly lower social interaction ratios during adulthood, regardless of the social condition after stress (*p* < 0.05).

### Experiment 2—Effects of Social Condition on AST Performance of Adult Mice Exposed to Early Adolescent Social Defeat

**Figure 5:**
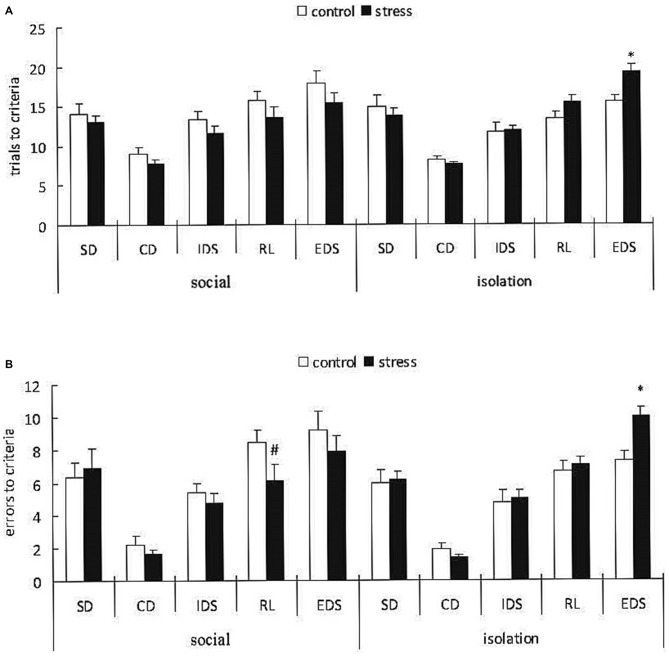
**Effects of social condition on early adolescent social defeat-induced impairment in cognitive flexibility in the AST in adult mice (Mean/SEM).** Trials to criteria **(A)**, errors before criteria **(B)** *p < 0.05 compared with the control group with isolation housing; ^#^*p* < 0.05 compared with the control group with social housing.

A 3-way ANOVA (stress × social condition × stage) of the trials to the criterion with repeated measurements over the stages indicated a significant main effect of stage (*F*_(4,148)_ = 52.276, *p* < 0.0001); however, there was no effect of stress (*F*_(1,37)_ = 0.571, *p* = 0.454) or social condition (*F*_(1,37)_ = 0.011, *p* = 0.985). However, there was a significant stress × social condition interaction (*F*_(1,37)_ = 5.733, *p* < 0.05; Figure 5A). A subsequent ANOVA for all task stages indicated a significant stress × social condition interaction only for the EDS stage (*F*_(1,37)_ = 5.62, *p* = 0.023). *Post hoc* comparisons for the EDS stage demonstrated that the adult mice exposed to social defeat during EA subsequently reared in isolation required more trials to the criterion compared with the corresponding control mice (*p* < 0.05), whereas there was no difference between the control and stressed groups with social housing after stress.

A 3-way ANOVA (stress × social condition × stage) of the errors before the criterion indicated a significant main effect of stage (*F*_(4,148)_ = 66.322, *p* < 0.0001) but not stress (*F*_(1,37)_ = 0.632, *p* = 0.432) or social condition (*F*_(1,37)_ = 0.041, *p* = 0.842). However, there were marginally significant stress × social condition × stage (*F*_(4,148)_ = 2.116, *p* = 0.082) and stress × social condition interactions (*F*_(1,37)_ = 3.412, *p* = 0.073; Figure 5B). A subsequent ANOVA for all task stages indicated a significant stress × social condition interaction for the RL (*F*_(1,37)_ = 4.78, *p* = 0.035) and EDS (*F*_(1,37)_ = 5.66, *p* = 0.022) stages. *Post hoc* comparisons for the RL stage demonstrated that the adult mice exposed to social defeat during adolescence and subsequently housed in groups exhibited fewer trials before the criterion compared with the corresponding controls (*p* < 0.05). *Post hoc* comparisons for the EDS stage indicated that the adult mice exposed to social defeat during adolescence and subsequently housed in isolation exhibited more trials before the criterion compared with the corresponding controls (*p* < 0.05).

## Discussion

The present study investigated the interacting effects of developmental stage and social condition on social defeat-induced social avoidance and cognitive flexibility. We determined that social defeat caused significant and lasting social avoidance of the defeat context in mice of different ages, regardless of the social conditions (isolation or social housing) following stress. In adult mice, social defeat induced a short-term impairment in RL when tested during the week following the stress, and this effect dissipated after 6 weeks. In contrast, early but not late adolescent social defeat with isolation housing impaired cognitive flexibility in EDS in adulthood but not during adolescence, and this effect was ameliorated by social housing after the stress. These findings suggest that the effects of social defeat on emotional behavior and cognitive function are differentially affected by the developmental stage and social condition. Notably, social defeat during EA had a delayed developmental effect on cognitive flexibility in adult mice, and social condition was an important intermediary factor in this process.

### Effects of Social Defeat on Social Avoidance in Mice of Different Ages

Social avoidance is a common measurement of specific anxiety to defeat contexts (Young, 2002). Social interaction ratios are often calculated to assess social avoidance to target animals to exclude disturbances that result from novel testing equipment and individual differences in locomotor activity (Ramos et al., 2008; Golden et al., 2011). Consistent with previous studies (Crews et al., 2007; Varlinskaya and Spear, 2008), the present study demonstrated that the adolescent mice in the control group exhibited increased locomotor activity in a novel testing environment (demonstrated by more distance traveled in the arena for the first 2.5 min session without CD-1 mice) compared with the adult controls (data not shown). Our results also demonstrated that stressed mice of different ages exhibited significant decreases in the social interaction ratio compared with the corresponding controls when tested both shortly after and 6 weeks later (however, the long-term social avoidance effect in the mice exposed as adults was only marginal). The social condition had no effect on the decrease in the social interaction ratio induced by social defeat during EA. These results are consistent with the findings of most previous studies in adult and adolescent animals. Studies of adult rodents have demonstrated that social avoidance induced by social defeat typically lasts longer than other behaviors irrelevant to the defeat experience, such as anhedonia or general anxiety in the elevated plus maze or open field (Buwalda et al., 2005; Vidal et al., 2011), and the behavioral alterations were maintained longer in isolated housing compared with social housing (de Jong et al., 2005; Buwalda et al., 2011; McCormick and Green, 2013). Exposure to adolescent social defeat with social housing also induces social avoidance during adulthood (Vidal et al., 2007; Burke et al., 2010; Huang et al., 2013). These findings suggest that social avoidance may comprise a reliable and long-lasting marker for defeat experiences during various conditions (e.g., animals of different ages and social conditions after stress, such as in this study).

### Effects of Social Defeat on AST Performance of Mice of Different Ages

The AST is a well-established model used to test different components of cognitive flexibility in rodents, particularly RL and EDS, which are specifically mediated by the mPFC and OFC, as well as their relevant pathways (Birrell and Brown, 2000; McAlonan and Brown, 2003; Ragozzino, 2007; Floresco et al., 2008). The present study demonstrated that social defeat exerts complex effects on cognitive flexibility depending on the developmental stage and social condition.

First, chronic social defeat induced a short-term impairment in RL in adult mice when tested during the week following stress. Various stressors have been demonstrated to impair cognitive flexibility as a result of structural and functional alterations in the corresponding PFC sections when tested shortly after stress (typically at approximately 1 week). For example, chronic restraint stress caused a selective deficit in EDS and a corresponding retraction of dendritic arbors in the mPFC (Liston et al., 2006). However, the cognitive deficit induced by adult social defeat was not identified after 6 weeks. This finding is consistent with a recent study in which social defeat-induced deficits in cognitive flexibility in adult rats also dissipated when tested after 5 weeks (Snyder et al., 2015). The reversibility of cognitive alterations induced by social defeat during adulthood may be related to the recovery of the corresponding changes in the PFC (Davidson and McEwen, 2012). For example, the morphological changes and synaptic loss in the PFC induced by stress in adulthood are reversible following a period of recovery (Radley and Morrison, 2005).

Second, the mice that experienced social defeat in EA but not LA and were housed in isolation exhibited cognitive dysfunction, especially in EDS during adulthood but not during adolescence. Similar delayed effects of stressor exposure in early life have also been reported in other studies (Toth et al., 2008; Shao et al., 2009). It is thought that the neural system that undergoes rapid development is sensitive, and early stress at this stage may alter the trajectories of critical brain developmental events, which results in the expression of behavioral symptoms after their maturation during adulthood (Andersen and Teicher, 2008). In rodents, significant remodeling of the PFC continues throughout childhood and adolescence (Spear, 2000; Giedd, 2008). Specifically, the structure of the mPFC and its connections with subcortical areas, such as the amygdala and hippocampus, undergo profound development with peak increases in synapses and receptor expression in pre- to EA and subsequent decreases by pruning to adult levels (Andersen, 2003). Thus, earlier pre- to EA, which comprises a period with rapid and marked developmental alterations in the mPFC, may represent a sensitive period for the effects of stress on the developmental trajectory and subsequent function of this area. Consistently, the deficit in EDS, which is specifically mediated by the mPFC, was stably induced by social defeat during EA in both experiments. There was a deficit in RL in the adult mice stressed during EA in Experiment 1; however, this result did not occur in Experiment 2. The discrepancy in RL may be related to a fine adjustment of social defeat parameters in the two experiments, including direct physical contact for 5 min in Experiment 1 compared with 3–5 min according to the intensity of the attack by the resident in Experiment 2 to reduce serious physical injury and the loss of stressed mice. Considering the specific effect of the OFC on RL, the results further suggested that the mPFC may comprise an area more sensitive to early adolescent stress compared with the OFC. There is a lack of literature that directly addresses this possibility; however, evidence has indicated that adolescent stress, particularly early adolescent stress, changes the synaptic density and morphology in the mPFC in adult animals (Leussis et al., 2008; Eiland et al., 2012). These structural abnormalities in the mPFC are associated with the deficit in EDS (Liston et al., 2006; Ragozzino, 2007).

Third, there was an interaction effect between early adolescent social defeat and social condition on cognitive dysfunction in adulthood, which was manifested by consistent impairment of EDS induced by early adolescent social defeat with isolation housing after stress; moreover, this effect was ameliorated by social housing (with siblings) after stress. Studies of humans have demonstrated that the potential consequences of being bullied during childhood and adolescence include social withdrawal and decreased social contacts, which are closely related to increased depression and anxiety in later life (Gaudin et al., 1993; Newman et al., 2005). In corroboration, the present study provided extensive evidence that the social condition modulates cognitive alterations in adulthood induced by social defeat during adolescence. The protective effects of social housing after defeat were also demonstrated by decreased errors before the criterion in RL in the stressed mice compared with the group-housed controls. However, these results are inconsistent with the recent findings of Snyder et al. (2015), who demonstrated that 5 days of social defeat during LA (PND 42–46) but not EA (PND 28–32) followed by social housing after the stress impaired the EDS performance in adult SD rats when tested after 5 weeks (Snyder et al., 2015). The performance of cognitive flexibility in the AST may be affected by many factors, including the animal species (Yuan et al., 2014), stress conditions (type and duration of stress; Liston et al., 2006; Danet et al., 2010; Luo et al., 2014), and age (Blakemore and Choudhury, 2006; Newman and McGaughy, 2011). In addition, a potentially stressful environment during social housing, including changing cage-mates and aggression among group-housed male mice, may exert detrimental effects on emotion and cognitive function (McQuaid et al., 2013; McCormick et al., 2015). To minimize these effects, the group-housed male mice comprised siblings and were maintained in the same group before and after the social defeat in the present study. Differences in the experimental designs may contribute to the discrepancies between our results and the results of Snyder et al. (2015).

## Conclusion

In humans, bullying during adolescence has previously been associated with an increased risk of the subsequent development of psychiatric disorders with emotional and cognitive symptoms. The present study used social defeat to model this link in mice. Our findings demonstrate that social defeat induces continuous context anxiety (social avoidance to the defeat context) in both adolescent and adult mice. However, only social defeat that occurred during EA caused a deficit in cognitive flexibility in adult mice, and the social condition after the stress appeared to play an important role in the development of the symptoms. Using the housing condition as an experimental variable to mimic different social contacts, e.g., the availability of social support that individuals may experience in daily life, the dependency of the cognitive effect on the housing condition in mice stressed during EA suggests that there may be potential therapeutic benefits to increasing social supports in adolescents. Further elucidation of the neurobiological substrates that underlie this link may indicate novel pharmacological targets for reversing stress-induced cognitive impairments.

## Author Contributions

WW designed the research; FZ and SY performed the research and acquired the data; FZ, SY and WW interpreted and analyzed the data; and FZ, SY, WW, and FS drafted, revised and wrote the manuscript.

## Conflict of Interest Statement

The authors declare that the research was conducted in the absence of any commercial or financial relationships that could be construed as a potential conflict of interest.
